# Role of sedation for agitated patients undergoing noninvasive ventilation: clinical practice in a tertiary referral hospital

**DOI:** 10.1186/s12890-015-0072-5

**Published:** 2015-07-13

**Authors:** Takeshi Matsumoto, Keisuke Tomii, Ryo Tachikawa, Kojiro Otsuka, Kazuma Nagata, Kyoko Otsuka, Atsushi Nakagawa, Michiaki Mishima, Kazuo Chin

**Affiliations:** Department of Respiratory Medicine, Kobe City Medical Center General Hospital, 2-1-1 Minatojima-minamimachi, Chuo-ku, Kobe, 650-0047 Japan; Department of Respiratory Medicine, Graduate School of Medicine, Kyoto University, 54 kawahara-cho, shogoin, sakyo-ku, Kyoto, 606-8507 Japan; Department of Respiratory Care and Sleep Control Medicine, Graduate School of Medicine, Kyoto University, 54 kawahara-cho, shogoin, sakyo-ku, Kyoto, 606-8507 Japan

**Keywords:** Continuous sedation, Intermittent sedation, Noninvasive ventilation, Agitation, Richmond Agitation Sedation Scale

## Abstract

**Background:**

Although sedation is often required for agitated patients undergoing noninvasive ventilation (NIV), reports on its practical use have been few. This study aimed to evaluate the efficacy and safety of sedation for agitated patients undergoing NIV in clinical practice in a single hospital.

**Methods:**

We retrospectively reviewed sedated patients who received NIV due to acute respiratory failure from May 2007 to May 2012. Sedation level was controlled according to the Richmond Agitation Sedation Scale (RASS). Clinical background, sedatives, failure rate of sedation, and complications were evaluated by 1) sedative methods (intermittent only, switched to continuous, or initially continuous) and 2) code status (do-not-intubate [DNI] or non-DNI).

**Results:**

Of 3506 patients who received NIV, 120 (3.4 %) consecutive patients were analyzed. Sedation was performed only intermittently in 72 (60 %) patients, was switched to continuously in 37 (31 %) and was applied only continuously in 11 (9 %). Underlying diseases in 48 % were acute respiratory distress syndrome/acute lung injury/severe pneumonia or acute exacerbation of interstitial pneumonia. In non-DNI patients (*n* = 39), no patient required intubation due to agitation with continuous sedation, and in DNI patients (*n* = 81), 96 % of patients could continue NIV treatment. PaCO_2_ level changes (6.7 ± 15.1 mmHg vs. -2.0 ± 7.7 mmHg, *P* = 0.028) and mortality in DNI patients (81 % vs. 57 %, *P* = 0.020) were significantly greater in the continuous use group than in the intermittent use group.

**Conclusions:**

According to RASS scores, sedation during NIV in proficient hospitals may be favorably used to potentially avoid NIV failure in agitated patients, even in those having diseases with poor evidence of the usefulness of NIV. However, with continuous use, we must be aware of an increased hypercapnic state and the possibility of increased mortality. Larger controlled studies are needed to better clarify the role of sedation in improving NIV outcomes in intolerant patients.

**Electronic supplementary material:**

The online version of this article (doi:10.1186/s12890-015-0072-5) contains supplementary material, which is available to authorized users.

## Background

Noninvasive ventilation (NIV) for acute respiratory failure is widely used; however, it is sometimes difficult to continue due to mask intolerance or inadequate cooperation. Antonelli et al. reported that 9 % of NIV users for acute respiratory failure had to be intubated for such reasons [1], and Carlucci et al. reported that the discontinuation rate of NIV due to patients’ refusal was up to 22 % [2]. Although NIV usage is not strictly indicated for agitated or uncooperative patients [3, 4], a questionnaire to pulmonologists and intensivists showed that 85 % of such patients had been sedated while under NIV, with 30 % receiving continuous sedation, suggesting its usefulness in clinical practice [5]. The efficacy of sedatives for agitated patients with acute respiratory failure undergoing NIV was reported [3, 6–10]. However, such patients usually had specific diseases with strong proven evidence of NIV’s usefulness and were treated in the ICU. In clinical practice, patients undergoing NIV treatment did not always have such diseases or were not always treated in an ICU.

In clinical practice, NIV introduction depends not only on underlying diseases but also on social conditions such as do-not-intubate (DNI) status. Therefore, NIV may be introduced to patients having diseases with little evidence of its usefulness. We previously reported the efficacy of NIV for life-threatening acute exacerbation of interstitial pneumonia or asthma attack [11, 12], for which the evidence level for its usefulness was not high [3].

We hypothesized that we could control agitated patients with sedation without severe complications regardless of evidence of NIV’s usefulness for their underlying diseases. Therefore, we retrospectively evaluated the efficacy and safety of sedation that was used intermittently or continuously for agitated patients during NIV treatment in clinical practice.

## Methods

### Patients

Our hospital is a 700-bed tertiary care center that plays a central role in treating emergency patients in the surrounding area. Among consecutive patients over 16 years old who underwent continuous NIV due to acute respiratory failure from May 2007 to May 2012, we retrospectively evaluated patients who received sedatives for agitation during NIV.

We assigned patients to 3 groups; one group received sedatives only intermittently (intermittent only), a second group was switched to continuous sedation after intermittent sedation (switched to continuous) and the third group was initially sedated continuously (initially continuous). According to code status, we also classified patients into non-DNI and DNI groups. Patients in the non-DNI group were intubated and mechanically ventilated if control was not achieved by NIV, while patients in the DNI group were continuously controlled by NIV and were not intubated even if consciousness deteriorated following sedation or their conditions became critical. Code status of neurologically incompetent patients was determined by discussion with relatives. When patients or their families did not want ventilation to be provided (including NIV) or their baseline status was difficult to maintain with NIV, we suggested that ventilation not be applied from the viewpoint of ethics.

This study was approved by our institutional review board (Institutional Review Board of Kobe City Medical Center General Hospital; 1304–1), and informed consent was waived.

### Noninvasive ventilation

NIV was started when 1) SpO_2_ was <90 % despite inhalation of oxygen >10 l/min via reservoir mask; 2) PaCO_2_ levels were >45 mmHg with acute respiratory acidosis; or 3) patients had signs of respiratory distress, including a respiratory rate >24 and increased accessory respiratory muscle use. Patients were managed with NIV in the ICU, emergency ward, or general ward by expert respiratory staff. Patients in a general ward were put in large separated rooms for intensive care and monitored 24 h per day. NIV was performed with a Drager ventilator (Carina; Drager, Lübeck, Germany) or Philips ventilator (Respironics V60 or Respironics BiPAP Vision; Philips, Andover, MA, USA) with the pressure support ventilation (PSV) mode or continuous positive airway pressure (CPAP) mode via a full face mask. The ventilator setting and selection of either the CPAP or PSV mode were generally determined based on the criteria for initiation of NIV described above. The PSV was selected if a patient met criterion 2) and/or 3), but if a patient had only hypoxemia and met criterion 1), we selected the CPAP mode. For the PSV mode, the initial setting was a respiratory rate of 12 breaths/min, inspiratory positive airway pressure of 10 cm H_2_O, and expiratory positive airway pressure of 4 cm H_2_O. For the CPAP mode, the first setting was a positive end expiratory pressure of 8 cm H_2_O. The FiO_2_ was adjusted to keep the SpO_2_ > 90 %. After the start of NIV treatment, NIV settings were modified by physicians proficient in NIV treatment according to each patient’s condition. At first NIV treatment was performed all day. However, we discontinued NIV treatment in the cases that met all the following criteria: 1) SpO_2_ was >90 % with the inhalation of oxygen <10 l/min via reservoir mask; 2) PaCO_2_ levels were <45 mmHg or patients did not suffer acute respiratory acidosis; and 3) patients had no signs of respiratory distress, including a respiratory rate >24 and increased accessory respiratory muscle use. When NIV treatment was not needed consecutively for 12 h, NIV treatment was considered to be finished.

### Sedatives

For intermittent use, risperidone or haloperidol was usually administered every 30–60 min by either a single dose or double dose (Table 1). For continuous use, either dexmedetomidine, midazolam, or propofol was the initial choice. Physicians in this hospital preferred to use a short-acting drug or a drug with a minimal respiratory depressant effect. When despite sedation dyspnea could not be controlled, we used morphine or fentanyl to alleviate the dyspnea.

**Table 1:** Initial dose and increasing and decreasing dose of each sedative drug

Drug	Initial dose	Increasing and decreasing dose
Risperidone	0.5 mg perorally	
Haloperidol	2.5–5 mg by intravenous infusion	
Dexmedetomidine	0.2 μg/kg/h by continuous intravenous infusion	0.1 μg/kg/h
Midazolam	0.03 mg/kg/h by continuous intravenous infusion	0.01 mg/kg/h
Propofol	0.3 mg/kg/h by continuous intravenous infusion	0.1 mg/kg/h
Morphine	0.02 mg/kg/h by continuous subcutaneous infusion	0.01 mg/kg/h
Fentanyl	0.05–0.1 μg/kg/h by continuous subcutaneous infusion	0.05 μg/kg/h

### Criteria for the beginning of sedation and administration of sedatives

When NIV was started according to the criteria described above, we used the Richmond Agitation Sedation Scale (RASS) [13] as an index of sedation for controlling agitation. Sedatives were administered when patients could not continue NIV due to agitation, and generally, +1 or more on the RASS was defined as an indication to administer sedation. Patients were most often managed between −2 and 0 on the RASS during sedation. Usually, sedation was initiated intermittently and if the target sedation level was not achieved, we began continuous administration. However, continuous sedation was introduced initially when physicians judged that intermittent sedation would not be sufficient to control agitation. At that time the attending physicians set the target range for the RASS, which was most often measured by medical staff. When the RASS deviated from the established range, the infusion rate was adjusted as shown in Table 1. When good control was not achieved with the first sedative, another was added.

### Outcome measures

We examined the clinical background, kinds of sedatives used, failure rate of sedation, and complications. All clinical and laboratory data were obtained from medical records. To assess severity of the respiratory failure, the PaO_2_/FiO_2_ (P/F) ratio at the initiation of NIV was calculated. Decision for intubation was left to attending physicians based on lack of control of agitation or progressive respiratory deterioration. In this study, failure of sedation consisted of the need for withdrawal of NIV because of absolute intolerance by patients despite the maximized analgo-sedative strategy. That is, in the non-DNI group, failure of sedation was declared when a patient was intubated due to agitation in spite of sedation, and failure of sedation in the DNI group was declared when NIV treatment could not be continued due to agitation. A RASS score of −4 or −5 indicated oversedation. Physiologic values were monitored and the RASS score, respiratory rate, heart rate, and blood pressure were checked before sedation and as closely as possible to 2 h, 6 h, and 24 h after the start of sedation. Arterial blood gas changes during 24 h following the initiation of sedation were also checked.

In measuring outcome, we compared differences in clinical background, 30-day mortality, and failure rate of sedation between the intermittent use group (intermittent only) and continuous use group (switched to continuous plus initially continuous groups combined) separately in the DNI and non-DNI groups.

### Statistical analysis

Continuous variables are expressed as mean ± standard deviation unless stated otherwise and were compared using the Mann–Whitney test. Categorical variables were compared using a chi-squared test or Fisher’s exact test, as appropriate. A *P*-value <0.05 was deemed statistically significant. All statistical analyses were performed using JMP 8.0.2 software (SAS Institute Inc., Cary, NC, USA).

## Results

### Study population

**Fig. 1:**
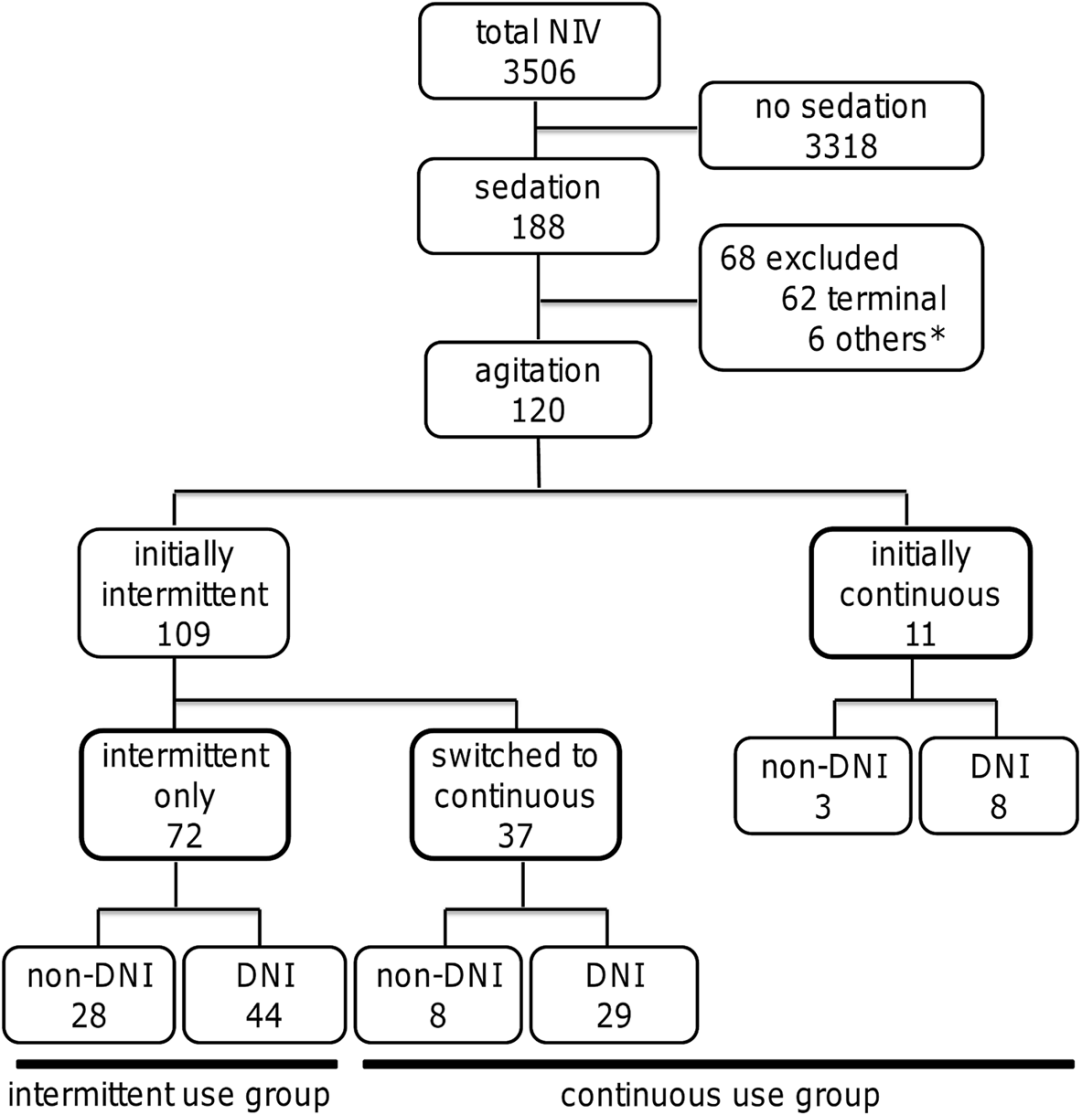
Number of patients at each stage of analysis and method of administration sedative drugs. Patients were categorized into the non-DNI group and DNI group. NIV, noninvasive ventilation; DNI, do-not-intubate. *: 4 patients used sedation for pain and 2 for convulsion

From May 2007 to May 2012, 3506 consecutive patients received NIV due to acute respiratory failure. Of these, 120 (3.4 %, non-DNI = 39; DNI = 81) patients were given sedatives to control agitation during NIV. Figure 1 shows the number of patients and method of administration of sedatives. Finally, sedation was performed only intermittently in 72 (60 %) patients, switched to continuously in 37 (31 %) and provided only continuously in 11 (9 %). The reasons for poor tolerance of NIV were mostly mask discomfort, pressure discomfort, or the combination of the two. Most expressions of poor tolerance occurred immediately after the start of NIV treatment.

### Underlying diseases

Table 2 shows underlying diseases of study patients. Of the 120 patients, 58 (48 %) had acute respiratory distress syndrome (ARDS)/acute lung injury (ALI)/severe pneumonia or acute exacerbation of interstitial pneumonia, diseases for which evidence of the usefulness of NIV was poor [3].

**Table 2:** Underlying diseases in each patient group

	Evidence level^a^	Intermittent only (*n* = 72)		Switched to continuous (*n* = 37)		Initially continuous (*n* = 11)		Total
		Non-DNI (*n* = 28)	DNI (*n* = 44)	Non-DNI (*n* = 8)	DNI (*n* = 29)	Non-DNI (*n* = 3)	DNI (*n* = 8)	*n* = 120
Acute exacerbation of COPD	1 (favorable)	0 (0 %)	4 (9 %)	0 (0 %)	0 (0 %)	0 (0 %)	0 (0 %)	4 (3 %)
Cardiogenic pulmonary edema	1 (favorable)	11 (39 %)	2 (5 %)	1 (13 %)	5 (17 %)	0 (0 %)	1 (13 %)	20 (17 %)
Acute respiratory failure in immunosuppressed state	1 (favorable)	5 (18 %)	3 (7 %)	3 (38 %)	4 (14 %)	2 (67 %)	0 (0 %)	17 (14 %)
Bronchial asthma	3 (favorable)	1 (4 %)	1 (2 %)	1 (13 %)	0 (0 %)	0 (0 %)	0 (0 %)	3 (3 %)
ARDS/ALI/severe pneumonia	2 or 3 (caution)	5 (18 %)	20 (45 %)	1 (13 %)	9 (31 %)	1 (33 %)	3 (38 %)	39 (33 %)
Acute exacerbation of interstitial pneumonia	4 (caution)	2 (7 %)	4 (9 %)	1 (13 %)	9 (31 %)	0 (0 %)	3 (38 %)	19 (16 %)
Sequela of pulmonary tuberculosis	NA	0 (0 %)	4 (9 %)	0 (0 %)	2 (7 %)	0 (0 %)	0 (0 %)	6 (5 %)
Others^b^	NA	4 (14 %)	6 (14 %)	1 (13 %)	0 (0 %)	0 (0 %)	1 (13 %)	12 (10 %)

*n* number of patients, *DNI* do-not-intubate, *ARDS* acute respiratory distress syndrome, *ALI* acute lung injury, *NA* not available

^a^evidence level from previous report 2; Each disease is classified as favorable or caution according to evidence level of use of NIV; 1 is the highest evidence level and 4 is the lowest

^b^includes hepatogenic pleural effusion, carcinomatous lymphangitis, pulmonary embolism, reexpansion pulmonary edema, and cryptogenic organizing pneumonia

### Sedatives

Table 3 shows the prescribed sedatives. Twenty-four (50 %) patients received a single drug and the remaining patients received more than one drug for continuous use. With the exception of risperidone or haloperidol, hydroxyzine, quetiapine, diazepam or perospirone was used intermittently.

**Table 3:** Sedative drugs administered to each patient group

	Intermittent only (*n* = 72)		Switched to continuous (*n* = 37)		Initially continuous (*n* = 11)		Total
	Non-DNI (*n* = 28)	DNI (*n* = 44)	Non-DNI (*n* = 8)	DNI (*n* = 29)	Non-DNI (n = 3)	DNI (n = 8)	*n* = 120
Risperidone	13 (46 %)	20 (45 %)	5 (63 %)	13 (45 %)			51 (43 %)
Haloperidol	20 (71 %)	35 (80 %)	8 (100 %)	24 (83 %)			87 (73 %)
Others	0 (0 %)	10 (23 %)	1 (13 %)	0 (0 %)			11 (9 %)
Dexmedetomidine			4 (50 %)	10 (34 %)	0 (0 %)	4 (50 %)	18 (15 %)
Midazolam			3 (38 %)	5 (17 %)	0 (0 %)	3 (38 %)	11 (9 %)
Propofol			3 (38 %)	10 (34 %)	1 (33 %)	2 (25 %)	16 (13 %)
Morphine			1 (13 %)	16 (55 %)	2 (67 %)	4 (50 %)	23 (19 %)
Fentanyl			3 (38 %)	6 (21 %)	1 (33 %)	1 (13 %)	11 (9 %)

Number (%) for each sedative drug reflects use of more than 1 drug per patient

*DNI* do-not-intubate

### Baseline characteristics

Baseline characteristics of the non-DNI and DNI groups in the intermittent use group or continuous use group are shown in Table 4. Within the non-DNI group, patients in the continuous use group (*n* = 11) were significantly younger than in the intermittent use group (*n* = 28) and baseline severity assessed by the P/F ratio did not differ between the two groups.

**Table 4:** Baseline characteristics in non-do not intubate (DNI) and DNI groups

	Non-DNI group (*n* = 39)			DNI group (*n* = 81)		
	Intermittent (*n* = 28)	Continuous (*n =* 11)	*P*-value	Intermittent (n = 44)	Continuous (*n* = 37)	*P*-value
Gender (male/female)	22/6	8/3	0.70	26/18	28/9	0.11
Age (y)	71.1 ± 10.9	60.5 ± 14.3	0.035	80.5 ± 8.1	74.9 ± 9.9	0.010
Duration of NIV (d)	4.5 ± 4.5	9.7 ± 11.7	0.044	7.0 ± 4.7	8.5 ± 5.3	0.12
Duration of continuous sedation (d)	−	7.7 ± 12.0	−	−	5.1 ± 3.2	−
Managing ward						
general ward	6/28 (21 %)	2/11 (18 %)	0.82	18/44 (41 %)	9/37 (24 %)	0.11
emergency ward	17/28 (61 %)	7/11 (64 %)	0.87	23/44 (52 %)	21/37 (57 %)	0.69
ICU	5/28 (18 %)	2/11 (18 %)	0.98	3/44 (7 %)	7/37 (19 %)	0.10
Respiratory failure (without/with hypercapnia)^a^	18/5	9/1	0.42	14/28	23/11	0.003
P/F ratio (mmHg)	114 ± 49	108 ± 62	0.49	148 ± 80	111 ± 51	0.032
NIV setting (CPAP/ PSV)	15/13	7/4	0.57	13/31	11/26	0.99

*P/F* PaO2/FiO2, *CPAP* continuous positive airway pressure, *PSV* pressure support ventilation

^a^6 patients in non-DNI group and 5 in the DNI group did not undergo blood gas examination

In the DNI group, patients in the continuous use group (*n* = 37) were also significantly younger than in the intermittent use group (*n* = 44). The proportion of patients with hypercapnia was significantly higher in the intermittent use group than in the continuous use group. P/F ratio was significantly lower in the continuous use group.

Thirty-four of the 109 (31 %) non-DNI or DNI patients in the initially intermittent group were managed in a general ward at first. Later 2 of these patients were transferred to the ICU for the initiation of continuous sedation with intensive monitoring.

### Mortality and failure rate of sedation

Mortality rate of the study participants and failure rate of sedation are shown in Table 5.

**Table 5:** Mortality rates and failure rates of sedation

	Non-DNI group (*n* = 39)			DNI group (*n* = 81)		
	Intermittent (*n* = 28)	Continuous (*n* = 11)	*P*-value	Intermittent (*n* = 44)	Continuous (*n* = 37)	*P*-value
Mortality	6/28 (21 %)	1/11 (9 %)	0.37	25/44 (57 %)	30/37 (81 %)	0.020
Total intubation	8/28 (29 %)	3/11 (27 %)	0.94			
Intubation due to agitation	2/28 (7 %)^a^	0/11 (0 %)	0.36			
Discontinuation of NIV due to agitation				2/44 (5 %)^b^	1/37 (3 %)^c^	0.66

^a^includes haloperidol in 2 patients

^b^includes risperidone in 1, haloperidol in 1 patient

^c^includes midazolam and morphine in 1 patient

In non-DNI patients, 30-day mortality and the total intubation rate did not differ significantly between the intermittent use and continuous use groups. No patient in the continuous use group required intubation due to agitation while 2 patients (7 %) in the intermittent use group required intubation due to sedation failure. After all, 2 of 36 patients with initially intermittent sedation were intubated without switching to continuous sedation due to their uncontrolled agitation.

Among DNI patients, 30-day mortality was higher in the continuous use group. Two of the 44 patients (5 %) in the intermittent use group and 1 of the 37 patients (3 %) in the continuous use group could not continue NIV due to persistent agitation; therefore, 78 of 81 (96 %) DNI patients could continue NIV with sedation. Overall, 115 of 120 (96 %) patients studied continued NIV despite agitation.

### Adverse events

As shown in Table 6, no patient vomited or developed aspiration pneumonitis during NIV treatment. Among the adverse events, 1 patient who had been prescribed midazolam became hypotensive requiring dopamine, 1 patient experienced delirium, and 1 patient developed ileus, which improved following the discontinuation of sedatives. Three patients who had hypercapnia before sedation exhibited drowsiness due to progressive hypercapnia, which improved following an increase in pressure support levels. Before and after the start of sedation, the RASS score, respiratory rate, heart rate, and systolic blood pressure did not differ significantly between intermittent and continuous use groups, nor did acute changes occur during the 24 h from the start of sedation (Additional file 1).

**Table 6:** Adverse events during sedation

	Intermittent (*n* = 72)	Continuous (*n* = 48)
Oversedation	haloperidol 1	midazolam 1, propofol 1
Hypotension		morphine 1^a^, midazolam 1^b^
Delirium		morphine 1
Ileus		fentanyl 1

^a^improved after discontinuation of sedatives

^b^needed dopamine

**Fig. 2:**
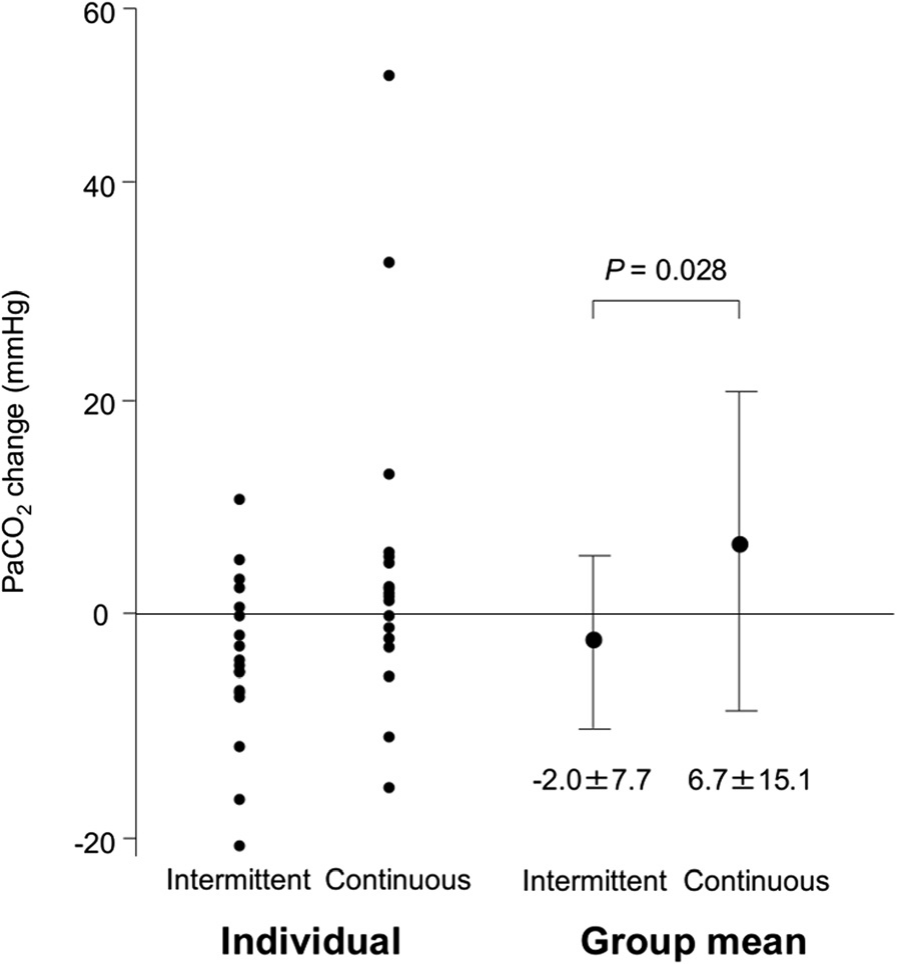
PaCO_2_ change within 24 h after initiation of each sedative. Individual data and group means are represented. Data are shown as mean ± standard deviation

The values of arterial blood gas were rechecked within 24 h from the start of sedation in 18 patients in the intermittent use group and 18 in the continuous use group. Changes in PaCO_2_ levels were significantly greater in the continuous use group than in the intermittent use group (Fig. 2). There were no significant differences in changes in pH and P/F ratio between groups (Additional file 2).

## Discussion

Of 3506 patients with acute respiratory failure administered NIV treatment in our institution, 120 (3.4 %) were sedated to control agitation. Fifty-eight (48 %) of the 120 patients had diseases for which there was not a high degree of evidence supporting NIV treatment such as ARDS, ALI, severe pneumonia, or acute exacerbation of interstitial pneumonia. However, no patient in the non-DNI group being administered continuous sedation required intubation due to agitation, and 96 % of patients in the DNI group were able to continue NIV treatment. Therefore, in clinical practice, we effectively used sedation to continue NIV in both DNI and non-DNI patients with management according to RASS scores. However, as to continuous use, we must be aware of an increased hypercapnic state and the possibility of increased mortality.

In this study, we found that by using several sedatives intermittently or continuously according to RASS scores, the NIV failure rate due to agitation was quite low (4 %). Previous studies have addressed the efficacy of sedation during NIV using dexmedetomidine [6–8], midazolam [8], propofol [9], and remifentanil [10] in patients with several diseases in which there was a high-to-intermediate level of evidence for NIV use. In addition, these patients were treated in an ICU. In this study, almost half of the patients had diseases with a low evidence level supporting NIV treatment (ARDS/ALI/severe pneumonia or acute exacerbation of interstitial pneumonia), and despite this, almost all were managed successfully with sedatives. Therefore, with the guidance of RASS scores, proficient medical teams for NIV treatment might control persistent agitation with appropriate sedatives while administering NIV, even in patients having diseases with poor evidence of the usefulness of NIV.

In this study, patients were divided into two groups; DNI and non-DNI groups. Although this resulted in a small sample size for analysis in some groups, we thought that differences in the usage of sedatives between DNI and non-DNI patients might be informative to those managing NIV treatment with sedatives. When NIV treatment is not effective in non-DNI patients, physicians usually choose intubation with mechanical ventilation. However, in DNI patients, intubation with mechanical ventilation is not performed when NIV treatment is not effective. That is, in the light of respiratory management, failure to control agitation would become fatal, and continuing NIV treatment with sedation is critical in the DNI group. On the other hand, in the non-DNI group, when we cannot continue NIV, we can perform intubation and continue mechanical ventilation. So in such cases we do not necessarily persist in continuing NIV treatment, and sedation is optional. In this study, 9 (23 %) non-DNI patients were intubated for reasons other than sedation insufficiency, such as exacerbation of the respiratory status or hemodynamic instability (Table 5). Therefore, especially in patients with underlying diseases in which there is not strong evidence for the effectiveness of NIV treatment, we should avoid delaying intubation due to persistence in administering sedatives during NIV in non-DNI patients [14, 15].

Among DNI patients, only 2 patients (5 %) in the intermittent use group and 1 patient (3 %) in the continuous use group discontinued NIV treatment, indicating that a high rate of persistence could be achieved with sedation. However, we must note that 30-day mortality in the DNI patients was higher in the continuous use group than in the intermittent use group. In previous reports, mortality was reported to be 44–57 % among DNI patients under NIV [16, 17] Also, among those with hypoxemic respiratory failure, the mortality rate of DNI patients was reported to be as high as 86 % [18]. In Japan when a patient cannot make decisions we usually provide NIV to those with a DNI status according to the family’s will, even when the baseline status is too poor for rescue or there is little evidence of NIV’s usefulness for the background disease. Many patients in the DNI group were severely ill and tended to become agitated and need sedation. Therefore, we often had to continue NIV with sedation as palliative care, which might on one hand contribute in some degree to the high mortality rate, and on the other hand contribute to prolonging useless agony. To avoid the latter, we discontinued NIV in DNI patients in accordance with patient’s and/or family’s decision in cases of persistent agitation. However, we must consider the possibility that the continuous sedation itself increased the mortality rate.

In this study, sedation during NIV treatment was introduced to 31 % of the study patients in the general wards, and in most of these patients treatment could be continued in the general wards. Many members of the medical staff of our hospital are highly experienced in NIV treatment so that NIV with sedatives could be controlled in general wards. However, as we did not have data on a sufficient number of patients to make a definitive conclusion on the safety of NIV treatment with sedatives, NIV treatment with sedatives should be applied cautiously and at present should be performed in an ICU.

As to complications, the change in the PaCO_2_ level within 24 h after initiation of sedation was significantly greater in the continuous use group than in the intermittent use group. This difference would be mainly due to the oversedated cases with hypercapnia, all of which had hypercapnia before sedation. However, their condition improved after increasing pressure support. Attention must be paid to the possibility of severe complications from continuous sedation such as hypotension or oversedation, especially in patients with hypercapnia prior to the start of sedation.

Our study had several limitations. First, it was retrospective and there was substantial heterogeneity in underlying diseases, sedation, therapies, and the sedatives used. However, the aim of this study was to clarify the role of sedation during NIV treatment in clinical practice, and we identified all consecutive patients using NIV to minimize selection bias. Second, the sample size was too small to detect significant differences. In addition, we could not compare the efficacy of each sedative or results according to each underlying disease due to the small number of patients. However, we could show the practical use of sedation during NIV treatment. Third, we could not directly compare sedated patients to unsedated patients who received NIV in the same study period. This makes it difficult to examine the cause of the high mortality rate among sedated patients in the DNI group. However, comparison with previous studies could have helped to evaluate the present results. Fourth, this study was conducted in a single institution that was proficient in the use of NIV treatment; therefore, we have to consider the indication for sedation because it depends on the proficiency or system in each institution. In consideration of these limitations, larger controlled studies are needed to better clarify the role of sedation in improving NIV outcomes in intolerant patients.

## Conclusions

Our results suggest that sedation during NIV can be used to enable continuation of NIV in agitated patients with either a DNI or non-DNI status with management according to RASS, even in patients with diseases for which there is little evidence of the usefulness of NIV. However, we must be aware of the possibility of an increased hypercapnic state and high mortality rate associated with continuous sedation, which may be due to the sedation itself. Also, continuing NIV under sedation is not appropriate in cases of failure to control agitation both in DNI patients in consideration of the risk of prolonging distress and agony, and in non-DNI patients considering the risk of unduly delaying intubation. In addition, it should be taken into consideration about the indication for sedation in each patient and the setting in which it is provided (general wards or ICU) because much depends on the proficiency or system in each institution.

## Additional files

Additional file 1:
**Changes in RASS score, respiratory rate, heart rate, and systolic blood pressure before and after the start of sedation. RASS, Richmond Agitation Sedation Scale.**
Additional file 2:
**Changes in pH and P/F ratio within 24 h after initiation of each sedative. P/F, PaO2/FiO2.** *Comparison of change in pH and PaO_2_/FiO_2_ ratio between intermittent and continuous use groups.

## Abbreviations

NIV: Noninvasive ventilation
DNI: Do-not-intubate
RASS: Richmond Agitation Sedation Scale
P/F: PaO_2_/FiO_2_
ARDS: Acute respiratory distress syndrome
ALI: Acute lung injury
